# Phytochromes A and B Mediate Light Stabilization of BIN2 to Regulate Brassinosteroid Signaling and Photomorphogenesis in *Arabidopsis*

**DOI:** 10.3389/fpls.2022.865019

**Published:** 2022-03-30

**Authors:** Jiachen Zhao, Guangqiong Yang, Lu Jiang, Shilong Zhang, Langxi Miao, Peng Xu, Huiru Chen, Li Chen, Zhilei Mao, Tongtong Guo, Shuang Kou, Hong-Quan Yang, Wenxiu Wang

**Affiliations:** ^1^Shanghai Key Laboratory of Plant Molecular Sciences, College of Life Sciences, Shanghai Normal University, Shanghai, China; ^2^School of Life Sciences, Fudan University, Shanghai, China

**Keywords:** *Arabidopsis*, phytochrome A (phyA), phytochrome B (phyB), brassinosteroid (BR), BRASSINOSTEROID-INSENSITIVE 2 (BIN2), BRI1-EMS SUPPRESSOR 1 (BES1), photomorphogenesis

## Abstract

Phytochromes A and B (phyA and phyB) are the far-red and red lights photoreceptors mediating many light responses in *Arabidopsis thaliana*. Brassinosteroid (BR) is a pivotal phytohormone regulating a variety of plant developmental processes including photomorphogenesis. It is known that phyB interacts with BES1 to inhibit its DNA-binding activity and repress BR signaling. Here, we show that far-red and red lights modulate BR signaling through phyA and phyB regulation of the stability of BIN2, a glycogen synthase kinase 3 (GSK3)-like kinase that phosphorylates BES1/BZR1 to inhibit BR signaling. The *BIN2* gain-of-function mutant *bin2-1* displays an enhanced photomorphogenic phenotype in both far-red and red lights. phyA-enhanced accumulation of BIN2 promotes the phosphorylation of BES1 in far-red light. *BIN2* acts genetically downstream from *PHYA* to regulate photomorphogenesis under far-red light. Both phyA and phyB interact directly with BIN2, which may promote the interaction of BIN2 with BES1 and induce the phosphorylation of BES1. Our results suggest that far-red and red lights inhibit BR signaling through phyA and phyB stabilization of BIN2 and promotion of BES1 phosphorylation, which defines a new layer of the regulatory mechanism that allows plants to coordinate light and BR signaling pathways to optimize photomorphogenesis.

## Introduction

Light serves as both the energy source for plant photosynthesis and critical environmental signal regulating plant growth and development (Jiao et al., 2007; Kami et al., 2010). Unlike animals, plants cannot perceive sunlight with their eyes, but they use a series of photoreceptors to perceive different qualitative and quantitative of ambient light. Among them, the far-red/red light photoreceptors phytochromes (PHYs, phyA to phyE), blue/UV-A light photoreceptors cryptochromes (CRYs, CRY1, and CRY2) and phototropins (PHOTs, PHOT1, and PHOT2), and UV-B light photoreceptor UV RESISTANCE LOCUS 8 (UVR8) are well studied in *Arabidopsis* (Cashmore et al., 1999; Briggs et al., 2001; Quail, 2002; Rizzini et al., 2011). These photoreceptors are involved in the light-dependent regulation of a variety of physiological responses, such as seedling photomorphogenesis, stomatal opening and development, photoperiodic flowering, and circadian rhythms (Ahmad and Cashmore, 1993; Guo et al., 1998; Deng and Quail, 1999; Yang et al., 2000; Kinoshita et al., 2001; Quail, 2002; Sullivan and Deng, 2003; Mao et al., 2005; Liu et al., 2008; Kang et al., 2009).

There are five phytochromes in *Arabidopsis* designated phyA to phyE. Among them, phyA mainly acts as the far-red light photoreceptor under far-red light to promote photomorphogenesis, while phyB is the primary red light photoreceptor that mediates red light-induced photomorphogenesis under red light (Quail, 2002). Phytochromes perceive light with the covalently attached linear tetrapyrrole chromophore and exist in two different conformations: the red light-absorbing Pr form and the far red light-absorbing Pfr form (Rockwell et al., 2006). Phytochromes are in the inactive Pr form in the cytoplasm when synthesized, and then translocate into the nucleus as the active Pfr form upon light irradiation (Kircher et al., 1999; Li et al., 2011). Once activated, they interact with a group of PHYTOCHROME-INTERACTING-FACTORS (PIFs), which are basic helix-loop-helix transcription factors and act as the key negative regulators in the phytochrome signaling cascade (Ni et al., 1998; Leivar and Quail, 2011). These interactions lead to the phosphorylation, ubiquitination and degradation of PIFs (Al-Sady et al., 2006; Shen et al., 2007, 2008), and eventually the activation or repression of their downstream target genes (Leivar and Quail, 2011). The RING-finger E3 ubiquitin ligase COP1 is the master negative regulator of light signaling, which interacts with and targets the degradation of many transcription factors in light signaling, such as HY5 and CONSTANS (CO), to control photomorphogenesis and flowering, respectively (Deng et al., 1992; Osterlund et al., 2000; Jang et al., 2008; Liu et al., 2008). Phytochromes interact with COP1, and its enhancer SPAs (SPA1–4), to repress the activity of COP1 and promote the photomorphogenesis (Jang et al., 2010; Lu et al., 2015; Sheerin et al., 2015). Cryptochromes and UVR8 regulate blue and UV-B light signaling by interacting with COP1/SPA1, respectively (Favory et al., 2009; Lian et al., 2011; Liu et al., 2011; Zuo et al., 2011). Moreover, cryptochromes mediate responses to low intensity blue light or high temperature by interacting with PIFs (Ma et al., 2016; Pedmale et al., 2016).

Brassinosteroids (BRs) are growth-promoting plant steroid hormones regulating many aspects of physiological processes of plants, including photomorphogenesis (Clouse, 2011; Lv and Li, 2020). BR is perceived by the receptor BRI1 (BRASSINOSTEROID INSENSITIVE1) and the co-receptor BAK1 (BRI1-ASSOCIATED RECEPTOR KINASE1), a membrane-localized receptor kinase complex, and then the activated BRI1 initiates the BR signal cascade through a series of phosphorylation and dephosphorylation events (Li and Chory, 1997; Li et al., 2002; Nam and Li, 2002; Kim et al., 2009; Zhu et al., 2013). BES1/BZR1 (BRI1-EMS SUPPRESSOR 1/BRASSINAZOLE-RESISTANT 1) are the key transcription factors of BR signaling, and the ratios of phosphorylated BES1/BZR1 (pBES1/pBZR1) to dephosphorylated BES1/BZR1 (dBES1/dBZR1) serve as the read-out of the BR signaling status (Wang et al., 2002; Yin et al., 2002). BIN2 (BRASSINOSTEROID INSENSITIVE2) is a GSK3-like protein kinase that acts as a negative factor in BR signaling to phosphorylate BES1/BZR1 and repress the expression of thousands of BR response genes (Li et al., 2001; Zhao et al., 2002), while protein phosphatase 2A acts as a positive regulator in BR signaling by dephosphorylating BES1/BZR1 (Tang et al., 2011).

Brassinosteroid is involved in inhibiting photomorphogenesis, as both BR biosynthesis and signaling deficient mutants display dwarf hypocotyl phenotype during photomorphogenesis (Chory et al., 1991; Li and Chory, 1997), and the gain-of-function mutant of *BIN2*, *bin2-1*, also displays a shortened hypocotyl phenotype in both darkness and light (Li et al., 2001). BIN2 integrates multiple signaling pathways by phosphorylating a range of proteins including the components in light signaling. For example, PIF3, PIF4, and PIF5 can be phosphorylated by BIN2 and then degraded via the 26S proteasome pathway to control transcription and hypocotyl growth (Bernardo-García et al., 2014; Ling et al., 2017). Meanwhile, light regulates the kinase activity of BIN2 through the COP1/SPA complex and HY5 (Ling et al., 2017; Li et al., 2020). Specifically, the COP1/SPA complex represses the activity of BIN2 and interferes with BIN2-PIF3 interaction in the dark to inhibit BIN2-mediated PIF3 destabilization (Ling et al., 2017), whereas HY5 enhances the activity of BIN2 via direct interaction in the light (Li et al., 2020). It is known that BR induced-degradation of BIN2 is dependent on the F-box protein KIB1 (Zhu et al., 2017), and that the repressor of BR signaling, the Nuclear Factor YCs (NF-YCs), interact with BIN2 to stabilize BIN2 (Zhang et al., 2021). However, whether light affects the stability of BIN2 to repress BR signaling is still unknown.

Previous studies have demonstrated that light represses BR signaling and promote photomorphogenesis through different mechanisms. For examples, both CRY1 and phyB interact with the dephosphorylated form of BES1 to inhibit its DNA-binding activity and repress BR signaling (Wang et al., 2018; Wu et al., 2019). In addition, CRY1 interacts with both BIN2 and BZR1 to bring them together and regulate hypocotyl elongation (He et al., 2019). Furthermore, phyB is shown to promote the amount of the phosphorylated BZR1 (Kim et al., 2014). In this study, we show that phyA and phyB repress BR signaling by inhibiting BR-induced degradation of BIN2 and promoting the phosphorylation of BES1. We demonstrate by protein-protein interaction studies that phyA and phyB interact directly with BIN2, which may promote the accumulation of BIN2 protein, as well as the interaction between BIN2 and BES1, leading to the phosphorylation of BES1. Our results provide a new insight into the molecular mechanism by which phyA and phyB regulate BR signaling and photomorphogenesis.

## Materials and Methods

### Plant Materials and Growth Conditions

Columbia (Col-0) seeds were used as the wild type (WT) of *Arabidopsis thaliana*. The *phyA*, *phyB*, *bin2-1* mutants, and the transgenic line overexpressing Flag-tagged BES1 (*BES1-Flag-OX*/WT), Myc-tagged phyB (*Myc-phyB-OX*/WT), YFP-tagged phyA (*phyA-YFP-OX*/WT) have been described previously (Wang et al., 2018; Wu et al., 2019; Xu et al., 2019). The *bin2-1* mutant was introgressed into *phyA* mutant background to generate *phyA bin2-1* double mutant. *BES1-Flag-OX*/WT was genetically crossed with *phyA* mutant to generate *BES1-Flag-OX*/*phyA*, respectively. These genotypes of plants were confirmed by phenotype analysis and/or protein expression assay. The primers in this study are all listed in Supplementary Table 1.

After the imbibed seeds were keep at 4°C for 3 days, they were grown on Murashige-Skoog (MS) nutrient medium plus 2% sucrose with 0.8% agar at 22°C under white light. The experiments involving red and far-red light illuminations were described previously (Wei et al., 2021). Light spectra and intensity were measured with a Hand Held spectroradiometer (ASD) and a Li250 quantum photometer (Li-Cor) (Wei et al., 2021).

### BIN2 Protein Degradation Assay in *Arabidopsis*

For the assays of the influence of phyA and phyB on BIN2 protein degradation in far-red and red lights, WT, *phyA* and *phyB* seedlings were grown in darkness, far-red or red light (1 μmol/m^2^/s or 50 μmol/m^2^/s) for 5 d. For the assays of the influence of far-red or red light exposure time on BIN2 protein degradation, WT and *phyA* or *phyB* seedlings were grown on MS plates supplemented with 2 μM BRZ (Sigma-Aldrich, USA) in darkness for 7 d, and then exposed to far-red (10 μmol/m^2^/s) or red light (100 μmol/m^2^/s) for 0, 5, 10, and 20 min, respectively. For the assay of the effects of far-red light intensity on the degradation of BIN2 protein, WT and *phyA* seedlings were grown in far-red light at the fluence rates of 0, 1, 3, and 10 μmol/m^2^/s for 5 d, respectively. For the assay of the effects of red light intensity on the degradation of BIN2 protein, WT and *phyB* seedlings were grown on MS plates supplemented with 2 μM BRZ in darkness for 7 d, and then exposed to red light at the fluence rates of 0, 50, 100, and 200 μmol/m^2^/s for 30 min, respectively. For the assays of the effects of phyA or phyB on brassinolide (BL)-induced degradation of BIN2, WT and *phyA* or *phyB* seedlings were grown on MS plates supplemented with 2 μM BRZ in darkness for 7 d, and then transferred into liquid MS medium containing 1 μM eBL (Sigma-Aldrich, USA) and exposed to far-red (10 μmol/m^2^/s) or red light (100 μmol/m^2^/s) for 30, 60, 90, and 120 min, respectively.

Lysis buffer containing 1 mM Pefabloc, cocktail and 50 μM MG132 was used to extract total protein, and Bradford assay (Bio-Rad, United States) was used to determine the total protein concentration. The supernatant of total protein was mixed with 5 × SDS loading buffer and boiled for 5 min, and subjected to Western blot analysis with an antibody against *Arabidopsis* BIN2 (Jiang et al., 2019).

### Measurements of Hypocotyl Length and Anthocyanin Content

The seeds used for hypocotyl length analysis were plated on MS medium with 2% sucrose and kept at 4°C for at 3 days, and then transferred to far-red or red light. Five-day-old seedlings were photographed with a digital camera (Nicon, Japan) (Xu et al., 2021). The hypocotyl lengths were measured using Image J software^1^ (Xu et al., 2021). Anthocyanin content was analyzed as described previously (Xu et al., 2021).

### BES1 Protein Phosphorylation Assay

For the assays of the influence of far-red and red lights exposure time on the phosphorylation of BES1 protein, *BES1-Flag-OX/*WT seedlings were grown in continuous darkness for 5 days, and then the dark-adapted seedlings were exposed to far-red or red light (10 μmol/m^2^/s or 50 μmol/m^2^/s) for different lengths of time (0, 1, 3, and 6 h). For the assay of the influence of phyA on the phosphorylation of BES1, *BES1-Flag-OX/*WT and *BES1-Flag-OX/phyA* seedlings were grown in darkness for 5 days, and then the dark-adapted seedlings were exposed to far-red light (10 μmol/m^2^/s) for different lengths of time (0, 1, 3, 6, 12, and 24 h). For the assays of the BIN2-dependent far-red and red lights regulation of the phosphorylation of BES1, *BES1-Flag-OX/*WT seedlings were grown on MS plates in darkness for 5 days, and then transferred into liquid MS supplemented with 10 mM LiCl, and kept in darkness or exposed to far-red or red light (10 μmol/m^2^/s or 50 μmol/m^2^/s) for different lengths of time (0, 1, 3, and 6 h), or *BES1-Flag-OX/*WT seedlings were grown on MS plates in darkness for 5 days, and then transferred into liquid MS supplemented with or without 1 μM BL or 10 mM LiCl for 3 h. For the assay of the BIN2-dependent phyA regulation of the phosphorylation of BES1, *BES1-Flag-OX/*WT and *BES1-Flag-OX/phyA* seedlings were grown on MS plates in darkness for 5 days, and then transferred into liquid MS medium containing 10 mM LiCl and kept in darkness or exposed to far-red light (10 μmol/m^2^/s) for different lengths of time (0, 1, 3, 6, 12, and 24 h).

Lysis buffer containing 1 mM Pefabloc, cocktail and 50 μM MG132 was used to extract total protein, and Bradford assay (Bio-Rad, United States) was used to determine the total protein concentration. The supernatant of total protein was mixed with 5 × SDS loading buffer and boiled for 5 min. An anti-Flag antibody (Sigma, F3165) was used to detect BES1-Flag protein.

### Pull-Down Assays

Pull-down assays were performed as described previously with minor modifications (Wang et al., 2018). pCold-TF-phyA-N (His-TF-phyA-N), pCold-TF-phyA-C (His-TF-phyA-C), pCold-TF-phyB-N (His-TF-phyB-N), and pCold-TF-phyB-C (His-TF-phyB-C) constructs were made previously (Wei et al., 2021). The DNA fragment of *BIN2* was cloned into pGEX-4T-1 (GE Healthcare Life Sciences, United States). His-TF-phyA-N, His-TF-phyA-C, His-TF-phyB-N, His-TF-phyB-C and GST-BIN2 fusion proteins were expressed in *E. coli* (Rosetta). For GST pull-down assays, MagneGST™ Glutathione Particles (Promega, United States, V8611) were used. Prey proteins were detected with anti-His (GenScript, China, A00186).

### Split-Luciferase Complementation Assays

The split luciferase complementation (split-LUC) assays were performed as described previously (Mao et al., 2020). The cDNA fragments encoding phyA, phyB, BIN2 and BES1 were cloned into pCambia1300-nLUC and pCambia1300-cLUC (Mao et al., 2020), respectively. These constructs were transformed into GV3101, respectively. GV3101 cells harboring the indicated combinations of constructs expressing nLUC- and cLUC-fused proteins were mixed at the ratio of 1:1 and infiltrated into tobacco (*Nicotiana benthamiana*) leaves (Mao et al., 2020).

To determine the effects of phyA and phyB on the interaction of BIN2 with BES1 via split-LUC assays, the cDNA fragments encoding phyA and GUS were cloned into vector pHB (Mao et al., 2005) to generate Myc-phyA and Myc-GUS. Cells harboring the constructs expressing cLUC-BES1 or BIN2-nLUC and Myc-phyA, Myc-phyB (Wu et al., 2019) or Myc-GUS (negative control) were mixed at a ratio of 1:1:1, and infiltrated into tobacco leaves, and then the LUC signal in tobacco leaves was collected by a luminescent imaging workstation after 2 days’ incubation with 1 mM D-luciferin sodium salt substrate.

### Semi-*in vivo* Pull-Down Assays

For the assays of the effects of far-red and red light on phyA-BIN2 and phyB-BIN2 interactions, GST-BIN2 bait protein and MagneGST™ Glutathione Particles were incubated together to make proteins-bound MagneGST™ Glutathione Particles, which were washed with lysis buffer (50 mM Tris-HCl, pH 7.5, 150 mM NaCl, 0.2% Trition-X-100) for three times. The prey protein phyA-YFP-containing extract was prepared from *phyA-YFP-OX* seedlings grown on white light (100 μmol/m^2^/s) for 4–5 days, and then adapted in darkness for 3 days, and finally remained in darkness for 30 min or exposed to far-red light (10 μmol/m^2^/s) for 30 min, while the prey protein phyB-YFP-containing extract was prepared from *Myc-phyB-OX* seedlings adapted to darkness for 5 days, and then remained in darkness for 30 min or exposed to red light (50 μmol/m^2^/s) for 30 min.

For the assays of the effects of phyA and phyB on BIN2-BES1 interaction, the prey protein extracts were prepared from *phyA*, *phyB*, and *phyA-YFP-OX* or *Myc-phyB-OX* seedlings grown on white light (100 μmol m^–2^ s^–1^) for 4–5 days, and then adapted in darkness for 3 days, and finally exposed to red (50 μmol/m^2^/s) or far-red light (10 μmol/m^2^/s) for 30 min. Lysis buffer containing 1 mM Pefabloc cocktail and 50 μM MG132 was used to homogenize seedlings. The extracts were centrifugated, and the supernatant was incubated with bait proteins-bound MagneGST™ Glutathione Particles for 1 h and washed for 4–5 times with 1 mL lysis buffer for each time, and then the precipitates were eluted into 25 μL SDS 1× loading buffer and subjected to Western blot analysis. Anti-Myc (Millipore, 05-724) or anti-GFP (Abmart, M20004H) antibody was used to detect Myc-phyB or phyA-YFP.

### Accession Numbers

Gene accession numbers used in this paper as follows: *phyA* (AT1G09570), *phyB* (AT2G18790), *BIN2* (AT4G18710), *BES1* (AT1G19350).

## Results

### phyA and phyB Are Involved in Mediating Far-Red and Red Lights Inhibition of Brassinolide-Induced Degradation of BIN2 Protein, Respectively

**FIGURE 1:**
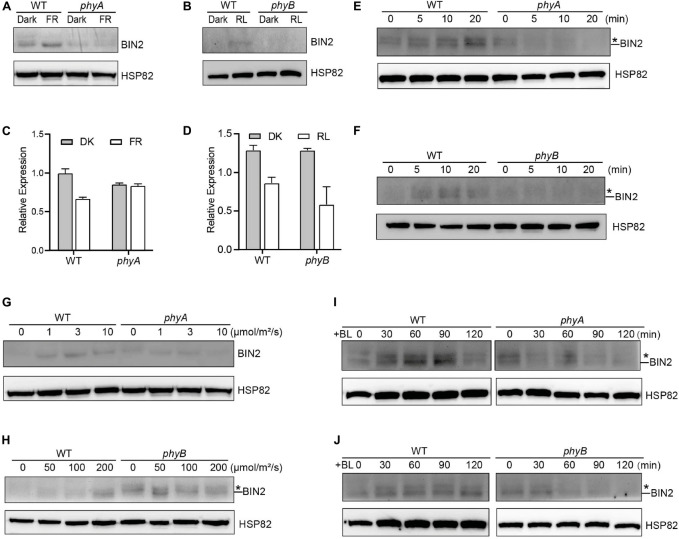
phyA and phyB Mediate Far-Red and Red lights Inhibition of BL-Induced Degradation of BIN2 Protein. **(A, B)** Western blotting assays showing phyA- and phyB-mediated far-red or red light inhibition of degradation of BIN2 protein. WT, *phyA* and *phyB* mutant seedlings were grown on MS plates in continuous darkness (DK) or far-red light (FR, 1 μmol/m^2^/s) **(A)** or red light (R, 50 μmol/m^2^/s) **(B)** for 5 d. **(C, D)** RT-qPCR assays showing the regulation of *BIN2* expression by phyA or phyB in **(A)** and **(B)**. Data correspond to the mean and standard deviation from three technical replicates. **(E, F)** Western blotting assays showing the effects of different exposure times of far-red or red light on the degradation of BIN2 protein. WT, *phyA* and *phyB* mutant seedlings were grown on MS plates supplemented with 2 μM BRZ in darkness for 7 d, and then exposed to far-red light (10 μmol/m^2^/s) **(E)** or red light (100 μmol/m^2^/s) **(F)** for the indicated lengths of time. **(G, H)** Western blot assays showing the effects of far-red or red light intensity on the degradation of BIN2 protein. WT and *phyA* mutant seedlings were grown on MS plates in far-red light at the fluence rates of 0, 1, 3, and 10 μmol/m^2^/s for 5 d, respectively **(G)**; WT and *phyB* mutant seedlings were grown on MS plates supplemented with 2 μM BRZ in darkness for 7 d, and then exposed to the indicated light intensities of red light **(H)** for 30 min. **(I, J)** Western blot assays showing the effects of phyA or phyB on the BL-induced degradation of BIN2 protein. WT, phyA and phyB mutant seedlings were grown on MS plates supplemented with 2 mM BRZ in darkness for 7 d, and then treated with 1 mM BL, and then exposed to far-red (10 μmol/m^2^/s) **(I)** or red light (100 μmol/m^2^/s) **(J)** for the indicated lengths of time. Asterisks shown in **(E)**, **(F)**, **(H)**, **(I)**, and **(J)** denote the possible modified BIN2 protein upon BRZ treatment.

The GSK3-like kinase BIN2 is the key negative regulator of BR signaling, and the regulation of BIN2 stability is important for BR signaling (Peng et al., 2008). Given our previous demonstrations that photoreceptors CRY1 and phyB inhibits auxin signaling by stabilizing the key auxin signaling repressors AUX/IAA proteins (Xu et al., 2018), and that CRY1 inhibits GA signaling by stabilizing the key GA signaling repressors DELLA proteins (Xu et al., 2021), we explored whether phyA and phyB might affect the stability of BIN2 to regulate BR signaling. To this end, we firstly performed immunoblot assays using an anti-BIN2 antibody to detect BIN2 protein level in WT, *phyA* and *phyB* mutant seedlings grown in continuous darkness or far-red or red light, respectively. The results showed that, in the WT background, much more BIN2 protein accumulated in far-red or red light than in the dark, whereas in the *phyA* or *phyB* mutant background, basically similar very low level of BIN2 was detected in the dark and far-red or red light (Figures 1A,B; Supplementary Figure 4). RT-qPCR analysis demonstrated that *BIN2* expression was not increased, but decreased to a varied extent in the WT or *phyA* or *phyB* mutant seedlings exposed to far-red or red light (Figures 1C,D). These results indicated that far-red and red lights induce the accumulation of BIN2 protein and that phyA and phyB might be responsible for mediating this process in far-red and red lights, respectively. As the BR biosynthesis inhibitor BRZ promotes BIN2 protein content in the etiolated seedlings (Peng et al., 2008), we then analyzed the accumulation of BIN2 protein using the BRZ-treated WT, *phyA* and *phyB* mutant seedlings grown in darkness for 7 d, and then exposed to different lengths of time of far-red and red lights, respectively. We found that BIN2, accumulated faster in WT than in *phyA* and *phyB* mutant backgrounds within 30 min far-red and red lights irradiation, respectively (Figures 1E,F; Supplementary Figure 4). We further analyzed BIN2 levels in the etiolated WT, *phyA* and *phyB* mutant seedlings exposed to different fluence rates offar-red and red lights, respectively. As WT and phyB mutants have low BIN2 protein levels when growing under continuous dark or red light, we treated WT and *phyB* mutant seedlings with 2 μM BRZ in darkness for 7 d, and then exposed to different fluence rates of red light for 30 min; While WT and *phyA* seedlings were grown in far-red light at the fluence rates of 0, 1, 3, and 10 μmol/m^2^/s for 5 d, respectively. The results showed that, in the WT background, BIN2 protein level increased as the fluence rate of far-red or red light increased (Figures 1G,H; Supplementary Figure 4), but hardly increased in *phyA* or *phyB* mutant background (Figures 1G,H; Supplementary Figure 4). Taken together, these results demonstrate that phyA and phyB mediate far-red and red light-induced accumulation of BIN2 protein, respectively.

Given the previous demonstration that the exogenous application of an active BR, brassinolide (BL) induces BIN2 degradation (Peng et al., 2008), we further examined whether phyA and phyB would inhibit the BL-induced degradation of BIN2 protein under far-red and red lights. To minimize the potential endogenous BRs content difference in WT and *phyA* or *phyB* mutant with different treatments, we applied BRZ in the subsequent assays. We performed Western blotting assay using the BRZ treated WT, *phyA* and *phyB* mutant seedlings grown in darkness for 5 d, and then transferred to liquid medium containing 1 μM eBL exposed to different lengths of time of far-red and red lights. As shown in Figures 1I,J and Supplementary Figure 4, the effects of red and far-red lights were stronger than those of BL within two hours of BL treatment, making BIN2 protein slightly increase in WT background as the light exposure time increased. Moreover, BIN2 protein was degraded much faster in *phyA* and *phyB* mutant seedlings than in WT seedlings when exposed to far-red and red lights. These results indicate that phyA and phyB are able to inhibit the BL-induced degradation of BIN2 protein under far-red and red lights.

## BIN2 Acts to Promote Photomorphogenesis in Far-Red and Red Lights

**FIGURE 2:**
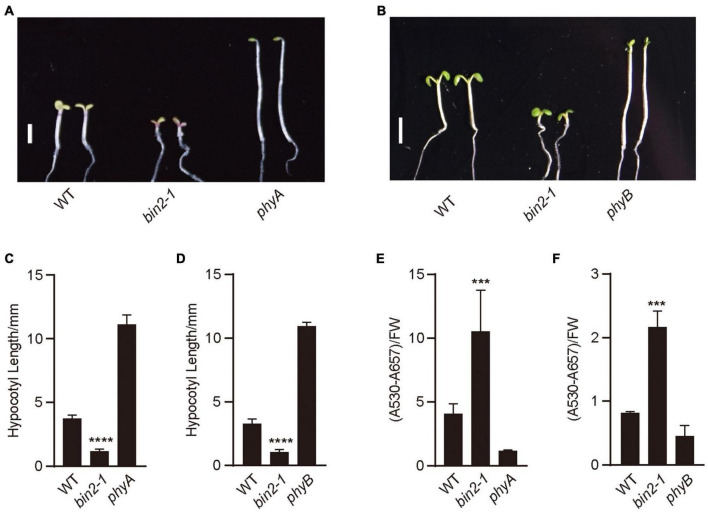
BIN2 acts to promote photomorphogenesis in red and far-red lights. **(A,B)** These genotypes of seedlings were grown under continuous far-red light (1 μmol/m^2^/s) **(A)** and red light (50 μmol/m^2^/s) **(B)** for 5 days, respectively. Scale bars, 2 mm. **(C,D)** Statistical analyses of hypocotyl lengths made from the genotypes of seedlings shown in **(A,B)**. Data are represented as mean ± SD (*n* ≥ 20). (Student’s *t*-test, ****P < 0.0001). **(E,F)** Anthocyanin contents analyses of the genotypes of seedlings shown in **(A,B)**. Data are represented as mean ± SD (*n* = 2). (Student’s *t*-test, ***P < 0.001).

To explore whether BIN2 might regulate photomorphogenic development in far-red and red lights, we examined the hypocotyl phenotype of the gain-of-function mutant of BIN2, *bin2-1* (Li et al., 2001), under red and far-red lights, respectively. The results showed that *bin2-1* seedlings developed significantly shorter hypocotyls than WT in far-red and red lights (Figures 2A–D). We then analyzed anthocyanin contents in *bin2-1* mutant grown in far-red and red lights, and found that *bin2-1* seedlings accumulated significantly more anthocyanin than WT in far-red or red light (Figures 2E,F). These results suggest that BIN2 is involved in promoting photomorphogenesis in far-red and red lights, respectively.

## phyA Promotes the Accumulation of the Phosphorylated BES1 in a BIN2-Dependent Manner Under Far-Red Light

**FIGURE 3:**
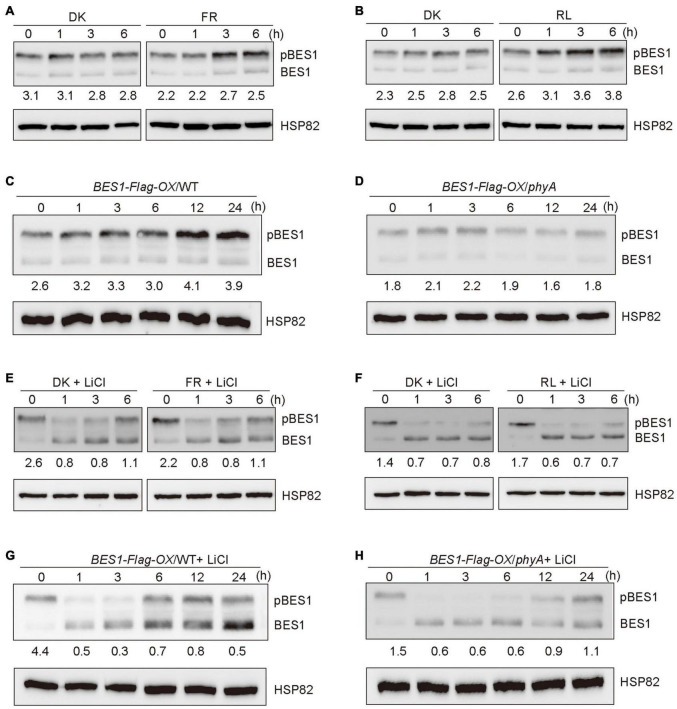
Phytochromes A mediates far-red light promotion of the phosphorylation of BES1 protein. **(A,B)** Western blot assays showing far-red and red lights promotion of phosphorylation of BES1-Flag protein. *BES1-Flag-OX*/WT seedlings were grown in darkness for 5 days, and then exposed to far-red light (FR, 10 μmol/m^2^/s) **(A)** or red light (R, 50 μmol/m^2^/s) **(B)** or adapted in the dark (DK) for the indicated lengths of time. In the Figure 3, pBES1 and BES1 denote the phosphorylated and dephosphorylated BES1, respectively. The ratio of phosphorylated to dephosphorylated was quantified using Image J and shown below each lane. **(C,D)** Western blot assays showing that phyA mediates far-red light promotion of phosphorylation of BES1-Flag protein. *BES1-Flag-OX*/WT **(C)** and *BES1-Flag-OX/phyA* **(D)** seedlings were grown in darkness for 5 days, and then exposed to the far-red light (10 μmol/m^2^/s) for the indicated lengths of time. **(E,F)** Western blot assays showing the effects of BIN2 on far-red and red lights promotion of the phosphorylation of BES1-Flag protein. *BES1-Flag-OX*/WT seedlings were grown on MS plates in darkness for 5 days, and then treated with 10 mM LiCl and exposed to far-red light (10 μmol/m^2^/s) **(E)** or red light (50 μmol/m^2^/s) **(F)** or adapted in the dark for the indicated lengths of time. **(G,H)** Western blot assays showing the effects of BIN2 on phyA-mediated far-red light promotion of the phosphorylation of BES1-Flag protein. *BES1-Flag-OX*/WT **(G)** and *BES1-Flag-OX/phyA* **(H)** seedlings were grown in darkness for 5 days, and then treated with 10 mM LiCl and exposed to far-red light (10 μmol/m^2^/s) for the indicated lengths of time.

It is known that BES1 and BZR1 are the important transcription factors of BR signaling, and the ratios of phosphorylated BES1/BZR1 (pBES1/pBZR1) to dephosphorylated BES1/BZR1 (dBES1/dBZR1) is the read-out of the BR signaling status (Wang et al., 2002). BIN2 phosphorylates BES1 and BZR1 to inhibit their DNA-binding activities, thus blocking the transduction of the BR signaling (Vert and Chory, 2006). Based on our demonstration that phyA mediates far-red light inhibition of the degradation of BIN2, we asked whether phyA might mediate far-red light modulation of the phosphorylation of BES1 to regulate BR signaling. To test this possibility, we firstly used the etiolated transgenic seedlings over-expressing BES1 fused to Flag in the WT background (*BES1-Flag-OX/*WT) (Wang et al., 2018) exposed to different lengths of time of far-red light, and examined the phosphorylation status of BES1. The results showed that the ratio of pBES1/dBES1 was increased as the far-red light exposure time increased (Figure 3A and Supplementary Figures 3, 4). We also found that red light is able to promote the accumulation of the phosphorylated of BES1, and the ratio of pBES1/dBES1 was also increased as the red light exposure time increased (Figure 3B and Supplementary Figures 3, 4). We then generated the transgenic seedlings overexpressing BES1-Flag in the *phyA* mutant background (*BES1-Flag-OX/phyA*), and analyzed the phosphorylation status of BES1 protein in *BES1-Flag-OX/*WT and *BES1-Flag-OX/phyA* seedlings adapted in the dark and then exposed to far-red light for different lengths of time, respectively. The results showed that the ratio of pBES1/dBES1 was increased faster in the WT background than in the *phyA* mutant background as the far-red light exposure time increased (Figures 3C,D and Supplementary Figures 3, 4). These results indicate that phyA mediates far-red light-induced accumulation of the phosphorylated BES1.

It is shown that LiCl inhibits the GSK3-like kinase activity of BIN2 and its homologs, leading to the accumulation of dephosphorylated BZR1/BES1 *in vivo* (Li et al., 2017). To further determine whether phyA would affect BES1 phosphorylation in a BIN2-dependent manner, we first performed immunoblotting analyses using *BES1-Flag-OX/*WT seedlings grown in darkness for 5 days, and then transferred to the liquid medium containing 10 mM LiCl adapted in darkness or exposed to far-red or red light for different lengths of time. The results showed that, in the presence of LiCl, the phosphorylated BES1 failed to accumulate as the far-red or red light exposure time increased, and the ratio of pBES1/dBES1 remained at a similar low level (Figures 3E,F and Supplementary Figures 3, 4). Moreover, in the presence of both LiCl and BL, the phosphorylated BES1 also hardly accumulated upon far-red or red light irradiation, and the ratio of pBES1/dBES1 was lower than in the presence of BL only or in the absent of both LiCl and BL (Supplementary Figures 1, 5). We then perform the same assays to evaluate the phosphorylation status of BES1 in the WT and *phyA* mutant backgrounds, respectively. The results showed that the treatment of LiCl reduced the accumulation of phosphorylated BES1 in both WT and *phyA* mutant backgrounds within 3 h (Figures 3G,H and Supplementary Figures 3–5). After 6 h treatment, the effects of LiCl became weak, and the ratio of pBES1/dBES1 increased faster in the WT than in the *phyA* mutant (Figures 3G,H and Supplementary Figures 3–5). After 24 h treatment, LiCl barely had effects, probably because the treatment was too long, which may trigger other processes affecting the phosphorylation of BES1 (Figures 3G,H and Supplementary Figures 3–5). Taken together, these results indicate that phyA-mediated far-red light promotion of the accumulation of the phosphorylated BES1 is dependent on BIN2.

## *BIN2* Acts Genetically Downstream of *PHYA* to Regulate Photomorphogenesis Under Far-Red Light

**FIGURE 4:**
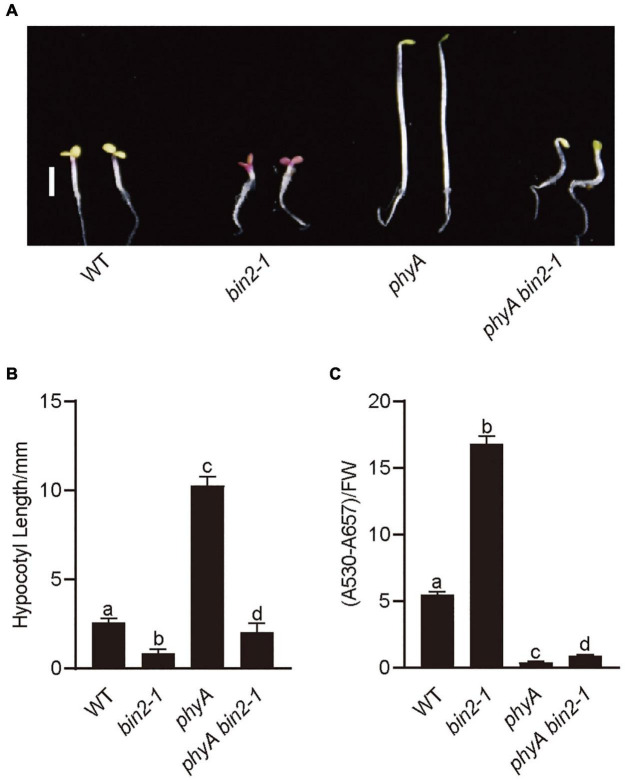
*BIN2* acts downstream from *PHYA* genetically to regulate photomorphogenesis. **(A)** Phenotypes of *bin2-1* and *phyA bin2-1* mutant seedlings grown in far-red light (1 μmol/m^2^/s) for 5 days. Scale bars, 2 mm. **(B)** Hypocotyl lengths and anthocyanin contents measurements in the genotypes of plants in **(A)**. Data in **(B)** is mean ± SD (*n* = 30). Data in **(C)** is mean ± SD (*n* = 2). Statistically significant differences are determined by a one-way analysis of variance (ANOVA), followed by Tukey’s least significant difference (LSD) test (*P* < 0.05), which are marked by letters “a” to “d.”

To explore the genetic relationships between *BIN2* and *PHYA*, we introgressed *bin2-1* into *phyA* mutant background by genetic crossing and generated the *phyA bin2-1* double mutant. Photomorphogenic phenotype analysis showed that the *phyA bin2-1* double mutant developed significantly shorter hypocotyls than *phyA* mutant in far-red light (Figures 4A,B), and accumulated more anthocyanin than *phyA* mutant under far-red light (Figure 4C). However, the *phyA bin2-1* double mutant developed a little longer hypocotyls, but produced much lower levels of anthocyanins than the *bin2-1* mutant. These results indicate that BIN2 may act as an important downstream component of phyA signaling to regulate hypocotyl elongation in far-red light, but play a minor role in mediating phyA signaling to regulate anthocyanin accumulation.

## Both phyA and phyB Physically Interact With BIN2

**FIGURE 5:**
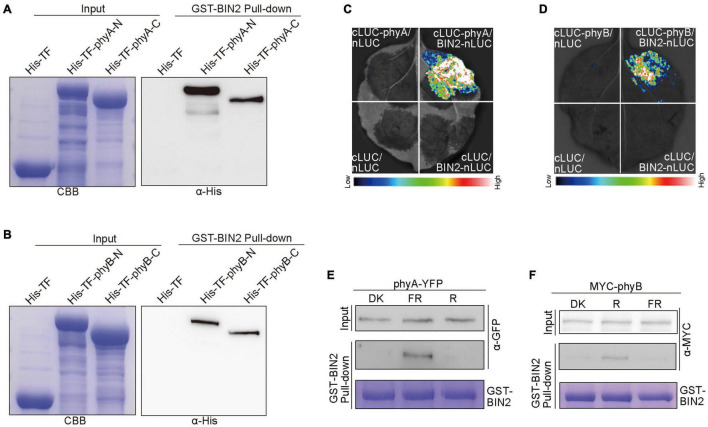
phyA and phyB interact with BIN2. **(A,B)** His pull-down assays showing the interactions of phyA-N, phyA-C **(A)**, phyB-N and phyB-C **(B)** with BIN2. GST-BIN2 served as a bait. His-TF-phyA-N, His-TF-phyA-C, His-TF-phyB-N, His-TF-phyB-C, and His-TF served as preys. The preys were detected with α-His antibody. **(C,D)** Split-LUC assays showing the interactions of phyA **(C)** and phyB **(D)** with BIN2. The vectors expressing nLUC and/or cLUC served as negative controls. **(E,F)** Semi-*in vivo* GST pull-down assays showing far-red and red light-dependent interactions of phyA **(E)** and phyB **(F)** with BIN2, respectively. GST-BIN2 served as a bait. The protein extracts prepared from *phyA-YFP-OX* and *Myc-phyB-OX* seedlings served as preys. *phyA-YFP-OX* and *Myc-phyB-OX* seedlings were grown in dark for 5 days, and then exposed to far-red light (FR, 10 μmol/m^2^/s) and red light (R, 50 μmol/m^2^/s) for 30 min, respectively.

Given our previous demonstrations that CRY1 or phyB inhibits auxin and GA signaling by interacting with AUX/IAA and DELLA proteins to stabilize them, respectively (Xu et al., 2018, 2021), we explored whether phyA and phyB might affect the stability of BIN2 by interacting with BIN2. To verify this assumption, we performed pull-down experiments with the recombinant His-TF-phyA-N (phyA N-terminus), His-TF-phyA-C (phyA C-terminus), His-TF-phyB-N (phyB N-terminus) and His-TF-phyB-C (phyB C-terminus) fusion proteins as preys, and GST-BIN2 as a bait. As shown in Figures 5A,B and Supplementary Figure 5, His-TF-phyA-N, His-TF-phyA-C, His-TF-phyB-N and His-TF-phyB-C were all pulled down by GST-BIN2, but not by the His-TF control, indicating that both the N and C termini of phyA and phyB interact with BIN2 protein *in vitro*.

To further prove whether phyA and phyB interact with BIN2 *in vivo*, we performed split luciferase complementation (split-LUC) assays in *Nicotiana benthamiana* leaves. phyA or phyB and BIN2 were fused to the N- and C-terminal halves of firefly luciferase (cLUC-phyA or cLUC-phyB and BIN2-nLUC), respectively. We found that the luciferase activity was reconstituted when cLUC-phyA or cLUC-phyB was co-expressed with BIN2-nLUC, but not when cLUC-phyA or cLUC-phyB was co-expressed with nLUC or when BIN2-nLUC was co-expressed with cLUC (Figures 5C,D). These results indicated that both phyA and phyB interact with BIN2 *in vivo*.

To investigate whether light quality may affect the interactions of phyA and phyB with BIN2, we performed semi-*in vivo* pull-down experiments. The recombinant GST-BIN2 protein was incubated with the protein extracts prepared from transgenic *Arabidopsis* seedlings overexpressing *phyA-YFP-OX* or *Myc-phyB-OX* that were dark-adapted before exposure to far-red or red light. As shown in Figures 5E,F and Supplementary Figure 5, BIN2 pulled down phyA and phyB from the extracts prepared from seedlings exposed to far-red and red lights, respectively, but not from those prepared from dark-adapted seedlings. These results suggest that phyA and phyB may interact with BIN2 in a far-red and red light-dependent manner, respectively.

## phyA and phyB May Promote the Interaction of BIN2 With BES1

**FIGURE 6:**
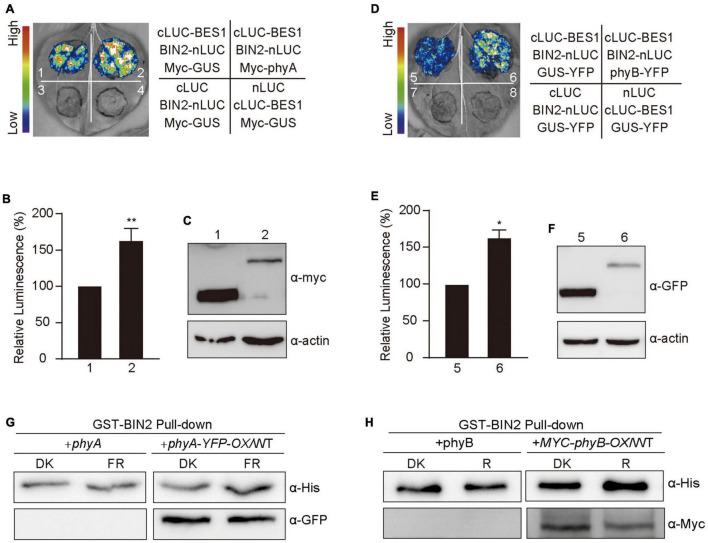
Phytochromes A and B promote the interaction of BIN2 and BES1 proteins. **(A–F)** Split-luciferase complementation imaging assays indicating phyA and phyB promotion of the interactions of BIN2 with BES1 in tobacco leaves. The luminescence intensity of the samples shown in **(A,D)** was quantitatively analyzed, respectively **(B,E)**. Data are represented as the mean of biological triplicates ± SD (*n* = 3). (Student’s *t*-test, *P < 0.05, **P < 0.01). The expression of Myc-phyA, Myc-phyB, and Myc-GUS (negative control) were detected with anti-Myc antibody **(C,F)**. **(G,H)** Cell-free GST pull-down assays showing phyA **(G)** and phyB **(H)** promotion of the interactions of BIN2 with BES1. GST-BIN2 served as bait. His-BES1 served as prey. The protein extracts prepared from the dark-adapted *phyA* and *phyA-YFP-OX*/WT or *phyB* and *Myc-phyB-OX*/WT seedlings exposed to far-red light (10 μmol/m^2^/s) or red light (50 μmol/m^2^/s) for 30 min, respectively.

Based on our demonstrations that phyA and phyB interact with BIN2 (Figure 5) and that phyA and phyB promote BIN2 accumulation and BES1 phosphorylation in far-red and red lights (Figures 1, 3), we explored whether phyA and phyB might promote the interaction of BIN2 with BES1. To do this, we first performed split-LUC assay in *N. benthamiana* leaves and confirmed the interaction between BIN2 with BES1 (Supplementary Figure 2A). We then detected the interaction strength between BIN2 and BES1 in the presence of Myc-phyA, Myc-phyB, and the control protein Myc-GUS, respectively. The results showed that the interaction of BIN2 with BES1 was enhanced significantly by phyA and phyB, but not by GUS (Figures 6A,B,D,E). Western blot assays showed that Myc-GUS was expressed at higher or similar levels compared with Myc-phyA and Myc-phyB (Figures 6C,F and Supplementary Figure 5). BIN2-nLUC and cLUC-BES1 were expressed at only slightly lower levels when co-expressed with Myc-GUS than when co-expressed with Myc-phyA (Supplementary Figures 2B, 5) or Myc-phyB (Supplementary Figures 2C, 5). These results indicate that phyA and phyB may promote the interaction of BIN2 with BES1.

We then performed cell-free pull-down assays with recombinant GST-BIN2, His-BES1 proteins and the protein extracts prepared from *phyA*, *phyB*, and *phyA-YFP-OX* or *Myc-*phyB*-OX* seedlings adapted in darkness and exposed to red and far-red light, respectively. We first tested this system by confirming the interaction between BIN2 and BES1, and found that the strength of the interaction between BIN2 and BES1 was enhanced as the amounts of His-BES1 increased (Supplementary Figures 2D, 5), which is consistent with the previous study (Zhao et al., 2002). We then added the protein extracts prepared from *phyA*, *phyB*, and *phyA-YFP-OX* or *Myc-phyB-OX* seedlings, and found that much more His-BES1 was pulled down by GST-BIN2 in the presence than in the absence of the activated phyA or phyB (Figures 6G,H and Supplementary Figure 5). These results indicate that phyA and phyB may promote the interaction of BIN2 with BES1 in a far-red and red light-dependent manner, respectively.

## Discussion

Given that the BR-deficient mutant *det2* (de-etiolated 2) was initially identified in the genetic screening for mutants showing constitutive photomorphogenic phenotype in darkness (Chory et al., 1991), it is likely that light and BR signaling are connected in some way. Through 30 years’ study, some impressive progresses have been made in dissecting the molecular mechanism of the crosstalk between light and BR signaling. It is known that BES1/BZR1 and BIN2 function at the heart of BR signaling networks, as BES1/BZR1 can directly regulate the expression of thousands of the downstream BR-responsive genes, and BIN2 can phosphorylate and destabilize BES1/BZR1 to repress BR signaling (Li et al., 2001; Wang et al., 2002; Yin et al., 2002; Vert and Chory, 2006). The multiple photoreceptors and the negative regulators of light signaling can mediate light repression of BR signaling by regulating the protein stability and the DNA-binding ability of BES1 and BZR1. Specifically, UVR8, phyB and CRY1 interact with dephosphorylated BES1 and BZR1 specifically to inhibit the DNA-binding ability of BES1 and repress the expression of their target genes (Liang et al., 2018; Wang et al., 2018; Wu et al., 2019). COP1 degrades the phosphorylated BZR1 in darkness, while SINAT E3 ligases are involved in degrading dephosphorylated BES1 protein in the light (Kim et al., 2014; Yang et al., 2017). It is shown that light regulates the kinase activity of BIN2 through the COP1/SPA1 complex and HY5 (Ling et al., 2017; Li et al., 2020). However, whether light regulates BR signaling through modulating the BIN2 protein stability is not known.

In this study, we report that phyA and phyB mediate light regulation of BR signaling and photomorphogenesis by modulating the stability of BIN2 protein. we show by protein expression assays that phyA- and phyB-mediated far-red and red lights signaling inhibits BL-induced degradation of BIN2 protein (Figure 1), and that the accumulated BIN2 promotes the phosphorylation of BES1 (Figure 3). Analysis of the phenotype of *bin2-1* mutant in monochromatic light conditions demonstrates that BIN2 acts to promote photomorphogenesis in far-red and red lights, respectively (Figure 2). Genetic interaction analysis indicates that BIN2 acts partially downstream of phyA to regulate photomorphogenesis under far-red light (Figure 4). We show by protein-protein interaction studies that phyA and phyB interact directly with BIN2 (Figure 5), and that phyA and phyB may promote the interaction between BIN2 and BES1 (Figure 6), which may in turn enhance the phosphorylation of BES1 by BIN2. These results, combined with the previous studies, suggest that phytochromes may mediate light inhibition of BR signaling through modulation of the activity of both BES1 and BIN2. On the one hand, they interact with the dephosphorylated BES1 to inhibit its DNA-binding ability (Wu et al., 2019); On the other hand, they stabilize BIN2 to promote the accumulation of the phosphorylated BES1, thus inhibiting its DNA-binding ability to promote photomorphogenesis (this report).

**FIGURE 7:**
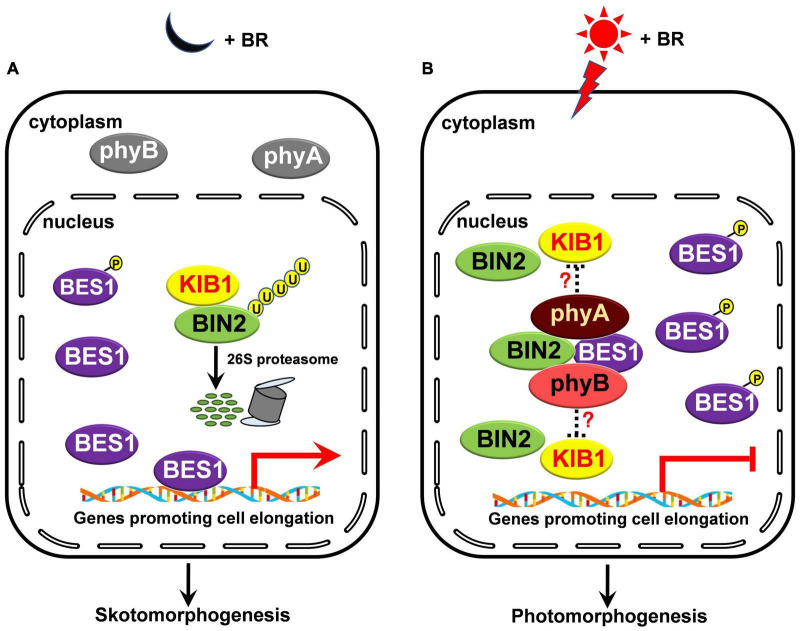
A model illustrating how phyA and phyB may inhibit BL-induced degradation of BIN2 protein and BR signaling. **(A,B)** In darkness and in the presence of BR, phyA and phyB are inactive in the cytoplasm and unable to interact with BIN2 and BES1 proteins. BIN2 is ubiquitinated by KIB1 and degraded through the 26S proteasome, thus BES1 is dephosphorylated, and able to bind to its target genes to promote their expression and skotomorphogenesis **(A)**; upon light irradiation, phyA and phyB are activated, and then translocated into the nucleus. We speculate that the interactions of phyA and phyB with BIN2 in light may promote the dissociation of BIN2 from its E3 ligase, such as KIB1, to stabilize BIN2 and enhance the interaction of BIN2 with BES1. Consequently, the phosphorylation of BES1 is promoted and its binding to its target genes is repressed, thus BR signaling is inhibited and photomorphogenesis is promoted **(B)**.

How do phyA and phyB regulate BIN2 protein stability? Previous studies have shown that phyB mediates red light induction of the degradation of EIN3 by enhancing the binding of the EBF1/EBF2 E3 ligase to EIN3 and regulate ethylene response (Shi et al., 2016), and that blue light-triggered interactions of CRY1 with AUX/IAA proteins or DELLA proteins inhibit the associations of SCF^TIR1^ with AUX/IAA proteins or the interactions of the GA receptor GID1 and SCF^SLY1^ with DELLA proteins to repress auxin or GA signaling (Xu et al., 2018, 2021). Given these demonstrations and our results showing that phyA and phyB interact with BIN2 directly (Figure 5), we speculate that the interactions of phyA and phyB with BIN2 may promote the dissociation of BIN2 from its E3 ligase, such as KIB1 (Zhu et al., 2017), to inhibit the ubiquitination and degradation of BIN2. Since it is known that NF-YCs promotes the autophosphorylation of BIN2 to protect BIN2 degradation, while the phosphorylated BSU1 dephosphorylates BIN2 to trigger the degradation of BIN2 via the 26S proteasome (Kim et al., 2009; Ryu et al., 2010; Zhang et al., 2021), it is also possible that the interactions of phyA and phyB with BIN2 may result in the inhibition of the dephosphorylation and degradation of BIN2. These possibilities will be worth exploring in future studies. In sum, we propose that, in darkness and in the presence of BR, as phyA and phyB are at their inactive Pr form, and localized in the cytoplasm, they cannot interact with BIN2 and BES1. BIN2 is ubiquitinated by KIB1 and degraded through the 26S proteasome, thus BES1 is dephosphorylated, and able to bind to its target genes to promote their expression and skotomorphogenesis (Figure 7A); Upon light irradiation, phyA and phyB are activated and translocate from the cytoplasm to the nucleus, where they may inhibit the association of BIN2 with KIB1 to stabilize BIN2 and enhance the interaction of BIN2 with BES1. As a result, BIN2 accumulates and the phosphorylation of BES1 is enhanced, and BES1 binding to its target genes is inhibited, leading to the inhibition of BR signaling and the eventual promotion of photomorphogenesis (Figure 7B).

## Data Availability Statement

The original contributions presented in the study are included in the article/Supplementary Material, further inquiries can be directed to the corresponding author/s.

## Author Contributions

WW and H-QY conceived the project and wrote the manuscript. JZ, GY, and WW designed the research plan. JZ and GY carried out the most of the experiments. All authors contributed the article and approved the submitted version.

## Conflict of Interest

The authors declare that the research was conducted in the absence of any commercial or financial relationships that could be construed as a potential conflict of interest.

## Publisher’s Note

All claims expressed in this article are solely those of the authors and do not necessarily represent those of their affiliated organizations, or those of the publisher, the editors and the reviewers. Any product that may be evaluated in this article, or claim that may be made by its manufacturer, is not guaranteed or endorsed by the publisher.

## Footnotes

1
http://rsbweb.nih.gov/ij/

## Supplementary Material

Supplementary Figure 1 Far-Red and Red Lights-Promoted Phosphorylation of BES1 is Dependent on BIN2. **(A,B)** Western blot assays showing the effects of BIN2 on far-red and red lights promotion of phosphorylation of BES1-Flag protein. *BES1-Flag-OX*/WT seedlings were grown on MS plates in darkness for 5 days, and then treated with or without 10 mM LiCl and 1 μM BL, and exposed to far-red light (FR, 10 μmol/m^2^/s) **(A)** or red light (R, 50 μmol/m^2^/s) **(B)** for 3 h.

Supplementary Figure 2 BES1 Interacts with BIN2 *in vivo* and *in vitro*. **(A)** Split-LUC assays showing the interactions of BES1 with BIN2. The vectors expressing nLUC and/or cLUC served as negative controls. **(B,C)** Western blot assays showing the protein levels of BES1-nLUC and cLUC-BIN2. The expression levels of BES1-nLUC and cLUC-BIN2 in Figure 6A **(B)** and Figure 6B **(C)** were detected with anti-LUC antibody. **(D)** Pull-down assay showing the interactions of BES1 with BIN2. GST-BIN2 served as bait, His-BES1 served as preys and detected with α-His antibody. 1×, 2×, 3×, 4× indicate the amounts of His-BES1.

Supplementary Figure 3 An additional biological replicate showing phyA mediating far-red light promotion of the phosphorylation of BES1 protein. **(A,B)** Western blot assays showing far-red and red lights promotion of phosphorylation of BES1-Flag protein. *BES1-Flag-OX*/WT seedlings were grown in darkness for 5 days, and then exposed to far-red light (FR, 10 μmol/m^2^/s) **(A)** or red light (R, 50 μmol/m^2^/s) **(B)** or adapted in the dark (DK) for the indicated lengths of time. In the Figure 3, pBES1 and BES1 denote the phosphorylated and dephosphorylated BES1, respectively. The ratio of phosphorylated to dephosphorylated was quantified using Image J and shown below each lane. **(C,D)** Western blot assays showing that phyA mediates far-red light promotion of phosphorylation of BES1-Flag protein. *BES1-Flag-OX*/WT **(C)** and *BES1-Flag-OX/phyA* **(D)** seedlings were grown in darkness for 5 days, and then exposed to the far-red light (10 μmol/m^2^/s) for the indicated lengths of time. **(E,F)** Western blot assays showing the effects of BIN2 on far-red and red lights promotion of the phosphorylation of BES1-Flag protein. *BES1-Flag-OX*/WT seedlings were grown on MS plates in darkness for 5 days, and then treated with 10 mM LiCl and exposed to far-red light (10 μmol/m^2^/s) **(E)** or red light (50 μmol/m^2^/s) **(F)** or adapted in the dark for the indicated lengths of time. **(G,H)** Western blot assays showing the effects of BIN2 on phyA-mediated far-red light promotion of the phosphorylation of BES1-Flag protein. *BES1-Flag-OX*/WT **(G)** and *BES1-Flag-OX/phyA* **(H)** seedlings were grown in darkness for 5 days, and then treated with 10 mM LiCl and exposed to far-red light (10 μmol/m^2^/s) for the indicated lengths of time.

Supplementary Figure 4 The original images of immunoblots used in this paper to prepare figures.

Supplementary Figure 5 The original images of immunoblots used in this paper to prepare figures.

Supplementary Table 1 The primers used in this study.
