# Corrigendum: Phytochromes A and B mediate light stabilization of BIN2 to regulate brassinosteroid signaling and photomorphogenesis in *Arabidopsis*

**DOI:** 10.3389/fpls.2022.1118271

**Published:** 2023-02-17

**Authors:** Jiachen Zhao, Guangqiong Yang, Lu Jiang, Shilong Zhang, Langxi Miao, Peng Xu, Huiru Chen, Li Chen, Zhilei Mao, Tongtong Guo, Shuang Kou, Hong-Quan Yang, Wenxiu Wang

**Affiliations:** ^1^ Shanghai Key Laboratory of Plant Molecular Sciences, College of Life Sciences, Shanghai Normal University, Shanghai, China; ^2^ School of Life Sciences, Fudan University, Shanghai, China

**Keywords:** *Arabidopsis*, phytochrome A (phyA), phytochrome B (phyB), brassinosteroid (BR), BRASSINOSTEROID-INSENSITIVE 2 (BIN2), BRI1-EMS SUPPRESSOR 1 (BES1), photomorphogenesis

In the published article, there was an error in **Figure 1** and Supplemental Figure 4. An antibody against a mammalian protein called BIN2 (Nouvsbio, NBP2-48690) to detect *Arabidopsis* BIN2 was used by mistake, which recognized *Arabidopsis* BIN2. The corrected **Figure 1** and Supplemental Figure 4 and their captions appear below:

**Figure 1:**
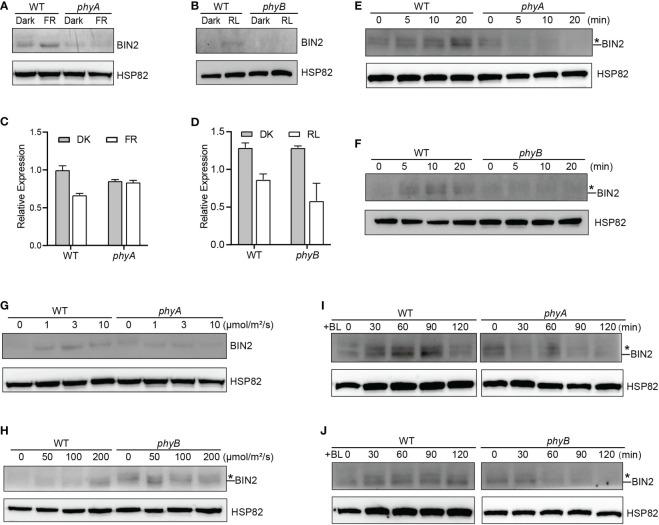
phyA and phyB Mediate Far-Red and Red lights Inhibition of BL-Induced Degradation of BIN2 Protein. **(A, B)** Western blotting assays showing phyA- and phyB-mediated far-red or red light inhibition of degradation of BIN2 protein. WT, *phyA* and *phyB* mutant seedlings were grown on MS plates in continuous darkness (DK) or far-red light (FR, 1 μmol/m^2^/s) **(A) ** or red light (R, 50 μmol/m^2^/s) **(B)** for 5 d. **(C, D)** RT-qPCR assays showing the regulation of *BIN2* expression by phyA or phyB in **(A)** and **(B)**. Data correspond to the mean and standard deviation from three technical replicates. **(E, F)** Western blotting assays showing the effects of different exposure times of far-red or red light on the degradation of BIN2 protein. WT, *phyA* and *phyB* mutant seedlings were grown on MS plates supplemented with 2 µM BRZ in darkness for 7 d, and then exposed to far-red light (10 μmol/m^2^/s) **(E)** or red light (100 μmol/m^2^/s) **(F)** for the indicated lengths of time. **(G, H)** Western blot assays showing the effects of far-red or red light intensity on the degradation of BIN2 protein. WT and *phyA* mutant seedlings were grown on MS plates in far-red light at the fluence rates of 0, 1, 3, and 10 µmol/m^2^/s for 5 d, respectively **(G)**; WT and *phyB* mutant seedlings were grown on MS plates supplemented with 2 µM BRZ in darkness for 7 d, and then exposed to the indicated light intensities of red light **(H)** for 30* min*. **(I, J)** Western blot assays showing the effects of phyA or phyB on the BL-induced degradation of BIN2 protein. WT, *phyA* and *phyB* mutant seedlings were grown on MS plates supplemented with 2 μM BRZ in darkness for 7 d, and then treated with 1 μM BL, and then exposed to far-red (10 μmol/m^2^/s) **(I)** or red light (100 μmol/m^2^/s) **(J)** for the indicated lengths of time. Asterisks shown in **(E)**, **(F)**, **(H)**, **(I)**, and **(J)** denote the possible modified BIN2 protein upon BRZ treatment.

In the published article there was an error in the **Materials and Methods**, *BIN2 Protein Degradation Assay in* Arabidopsis, paragraph 1 and 2. Following the correction to **Figure 1** and Supplemental Figure 4, the **Materials and Methods** needs to be updated accordingly.

The corrected paragraph appears below:

“For the assays of the influence of phyA and phyB on BIN2 protein degradation in far-red and red lights, WT, *phyA* and *phyB* seedlings were grown in darkness, far-red or red light (1 µmol/m^2^/s or 50 µmol/m^2^/s) for 5 d. For the assays of the influence of far-red or red light exposure time on BIN2 protein degradation, WT and *phyA* or *phyB* seedlings were grown on MS plates supplemented with 2 µM BRZ (Sigma-Aldrich, USA) in darkness for 7 d, and then exposed to far-red (10 µmol/m^2^/s) or red light (100 µmol/m^2^/s) for 0, 5, 10, and 20 min, respectively. For the assay of the effects of far-red light intensity on the degradation of BIN2 protein, WT and *phyA* seedlings were grown in far-red light at the fluence rates of 0, 1, 3, and 10 µmol/m^2^/s for 5 d, respectively. For the assay of the effects of red light intensity on the degradation of BIN2 protein, WT and *phyB* seedlings were grown on MS plates supplemented with 2 µM BRZ in darkness for 7 d, and then exposed to red light at the fluence rates of 0, 50, 100, and 200 µmol/m^2^/s for 30 min, respectively. For the assays of the effects of phyA or phyB on brassinolide (BL)-induced degradation of BIN2, WT and *phyA* or *phyB* seedlings were grown on MS plates supplemented with 2 µM BRZ in darkness for 7 d, and then transferred into liquid MS medium containing 1 µM eBL (Sigma-Aldrich, USA) and exposed to far-red (10 µmol/m^2^/s) or red light (100 µmol/m^2^/s) for 30, 60, 90, and 120 min, respectively.”

“Lysis buffer containing 1 mM Pefabloc, cocktail and 50 µM MG132 was used to extract total protein, and Bradford assay (Bio-Rad, United States) was used to determine the total protein concentration. The supernatant of total protein was mixed with 5 × SDS loading buffer and boiled for 5 min, and subjected to Western blot analysis with an antibody against *Arabidopsis* BIN2 (Jiang et al., 2019).”

In the published article there was an error in the **Results**, *phyA and phyB Are Involved in Mediating Far-Red and Red Lights Inhibition of Brassinolide-Induced Degradation of BIN2 Protein, Respectively*, paragraphs 1 and 2. Following the correction to **Figure 1** and Supplemental Figure 4, the **Results** needs to be updated accordingly.

The corrected paragraphs appear below:

“The GSK3-like kinase BIN2 is the key negative regulator of BR signaling, and the regulation of BIN2 stability is important for BR signaling (Peng et al., 2008). Given our previous demonstrations that photoreceptors CRY1 and phyB inhibits auxin signaling by stabilizing the key auxin signaling repressors AUX/IAA proteins (Xu et al., 2018), and that CRY1 inhibits GA signaling by stabilizing the key GA signaling repressors DELLA proteins (Xu et al., 2021), we explored whether phyA and phyB might affect the stability of BIN2 to regulate BR signaling. To this end, we firstly performed immunoblot assays using an anti-BIN2 antibody to detect BIN2 protein level in WT, *phyA* and *phyB* mutant seedlings grown in continuous darkness or far-red or red light, respectively. The results showed that, in the WT background, much more BIN2 protein accumulated in far-red or red light than in the dark, whereas in the *phyA* or *phyB* mutant background, basically similar very low level of BIN2 was detected in the dark and far-red or red light (**Figures 1A, B**; Supplementary Figure 4). RT-qPCR analysis demonstrated that *BIN2* expression was not increased, but decreased to a varied extent in the WT or *phyA* or *phyB* mutant seedlings exposed to far-red or red light (**Figures 1C, D**). These results indicated that far-red and red lights induce the accumulation of BIN2 protein and that phyA and phyB might be responsible for mediating this process in far-red and red lights, respectively. As the BR biosynthesis inhibitor BRZ promotes BIN2 protein content in the etiolated seedlings (Peng et al., 2008), we then analyzed the accumulation of BIN2 protein using the BRZ-treated WT, *phyA* and *phyB* mutant seedlings grown in darkness for 7 d, and then exposed to different lengths of time of far-red and red lights, respectively. We found that BIN2, accumulated faster in WT than in *phyA* and *phyB* mutant backgrounds within 30 min far-red and red lights irradiation, respectively (**Figures 1E, F**; Supplementary Figure 4). We further analyzed BIN2 levels in the etiolated WT, *phyA* and *phyB* mutant seedlings exposed to different fluence rates of far-red and red lights, respectively. As WT and phyB mutants have low BIN2 protein levels when growing under continuous dark or red light, we treated WT and *phyB* mutant seedlings with 2 µM BRZ in darkness for 7 d, and then exposed to different fluence rates of red light for 30 min; While WT and *phyA* seedlings were grown in far-red light at the fluence rates of 0, 1, 3, and 10 µmol/m^2^/s for 5 d, respectively. The results showed that, in the WT background, BIN2 protein level increased as the fluence rate of far-red or red light increased (**Figures 1G, H**; Supplementary Figure 4), but hardly increased in *phyA* or *phyB* mutant background (**Figures 1G, H**; Supplementary Figure 4). Taken together, these results demonstrate that phyA and phyB mediate far-red and red light-induced accumulation of BIN2 protein, respectively.”

“Given the previous demonstration that the exogenous application of an active BR, brassinolide (BL) induces BIN2 degradation (Peng et al., 2008), we further examined whether phyA and phyB would inhibit the BL-induced degradation of BIN2 protein under far-red and red lights. To minimize the potential endogenous BRs content difference in WT and *phyA* or *phyB* mutant with different treatments, we applied BRZ in the subsequent assays. We performed Western blotting assay using the BRZ treated WT, *phyA* and *phyB* mutant seedlings grown in darkness for 5 d, and then transferred to liquid medium containing 1 μM eBL exposed to different lengths of time of far-red and red lights. As shown in **Figures 1I, J** and Supplementary Figure 4, the effects of red and far-red lights were stronger than those of BL within two hours of BL treatment, making BIN2 protein slightly increase in WT background as the light exposure time increased. Moreover, BIN2 protein was degraded much faster in *phyA* and *phyB* mutant seedlings than in WT seedlings when exposed to far-red and red lights. These results indicate that phyA and phyB are able to inhibit the BL-induced degradation of BIN2 protein under far-red and red lights.”

The authors apologize for these errors and state that this does not change the scientific conclusions of the article in any way. The original article has been updated.

