# Supplementary material for: Phytochromes A and B Mediate Light Stabilization of BIN2 to Regulate Brassinosteroid Signaling and Photomorphogenesis in Arabidopsis
Source: Front Plant Sci. 2022 Mar 30;13:865019. doi: 10.3389/fpls.2022.865019 (PMC9005995; doi:10.3389/fpls.2022.865019)
Supplement: Supplementary Figure 1 — Far-Red and Red Lights-Promoted Phosphorylation of BES1 is Dependent on BIN2. (A,B) Western blot assays showing the effects of BIN2 on far-red and red lights promotion of phosphorylation of BES1-Flag protein. BES1-Flag-OX/WT seedlings were grown on MS plates in darkness for 5 days, and then treated with or without 10 mM LiCl and 1 μM BL, and exposed to far-red light (FR, 10 μmol/m2/s) (A) or red light (R, 50 μmol/m2/s) (B) for 3 h. [file Data_Sheet_1.doc]

**Phytochromes A and B Mediate Light Stabilization of BIN2 to Regulate Brassinosteroid Signaling and Photomorphogenesis in *Arabidopsis***

Jiachen Zhao^1,3^, Guangqiong Yang^1,3^, Lu Jiang^1^, Shilong Zhang^1^, Langxi Miao^2^, Peng Xu^2^, Huiru Chen^1^, Li Chen^1^, Zhilei Mao^1^, Tongtong Guo^1^, Shuang Kou^1^, Hong-Quan Yang^1^ and Wenxiu Wang ^1^,*

^1^ Shanghai Key Laboratory of Plant Molecular Sciences, College of Life Sciences, Shanghai Normal University, Shanghai 200234, China

^2^ School of Life Sciences, Fudan University, Shanghai 200438, China

^3^These authors contribute equally to this work

*Author for correspondence: Wenxiu Wang (Email: [wangwenxiu85@shnu.edu.cn](mailto:wangwenxiu85@shnu.edu.cn))

**Supplemental Figure 1. Far-Red and Red Lights-Promoted Phosphorylation of BES1 is Dependent on BIN2.**

**
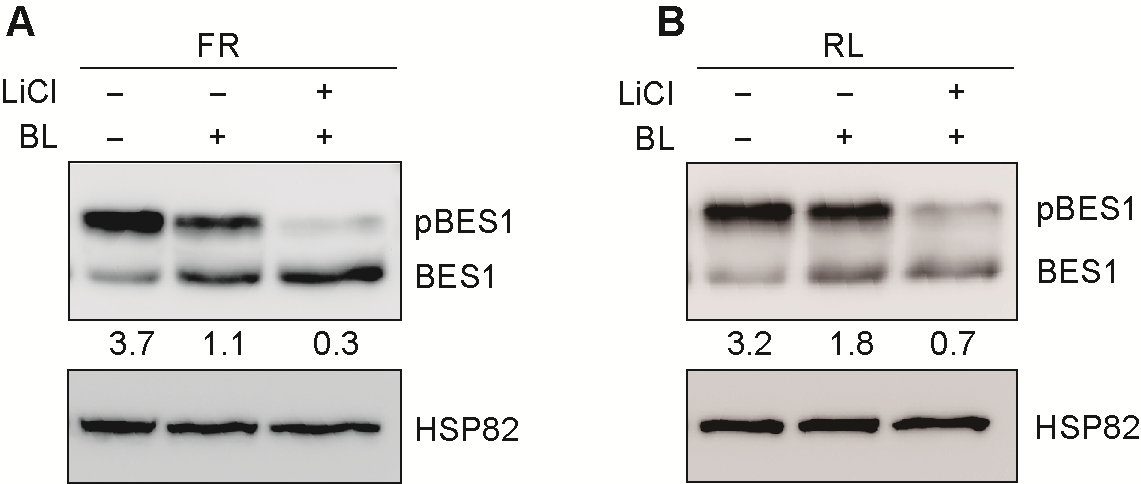
**

**(A,B)** Western blot assays showing the effects of BIN2 on far-red and red lights promotion of phosphorylation of BES1-Flag protein. *BES1-Flag-OX*/WT seedlings were grown on MS plates in darkness for 5 d, and then treated with or without 10 mM LiCl and 1 μM BL, and exposed to far-red­ light (FR, 10 μmol/m^2^/s) **(A)** or red­ light (R, 50 μmol/m^2^/s) **(B)** for 3 h.

**
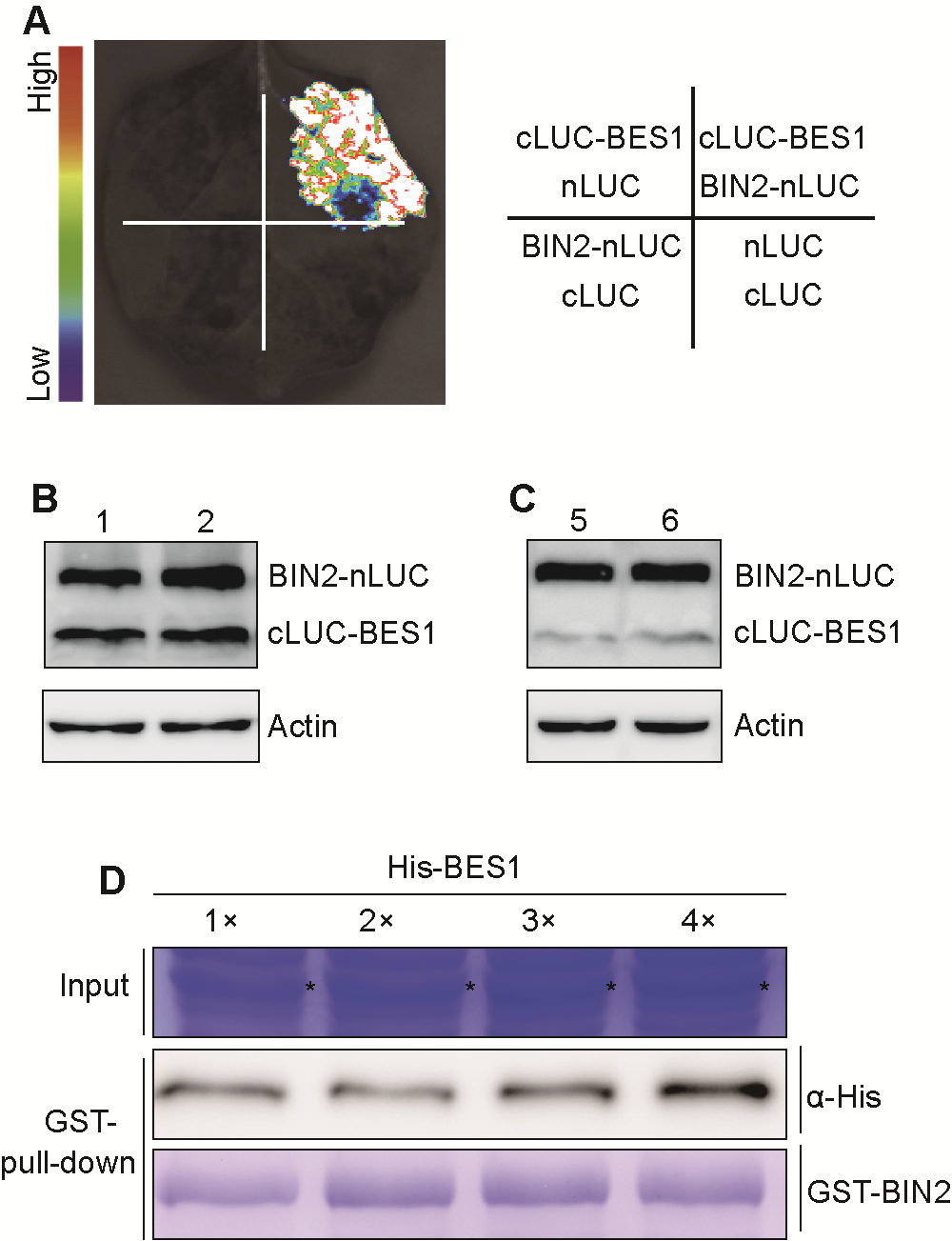
**

**Supplemental Figure 2. BES1 Interacts with BIN2 *in Vivo* and in *Vitro*.**

**(A)** Split-LUC assays showing the interactions of BES1 with BIN2. The vectors expressing nLUC and/or cLUC served as negative controls.

**(B,C)** Western blot assays showing the protein levels of BES1-nLUC and cLUC-BIN2. The expression levels of BES1-nLUC and cLUC-BIN2 in Figure 6A (**B**) and 6B (**C**) were detected with anti-LUC antibody.

**(D)** Pull-down assay showing the interactions of BES1 with BIN2. GST-BIN2 served as bait, His-BES1 served as preys and detected with α-His antibody. 1×, 2×, 3×, 4× indicate the amounts of His-BES1.

**Supplemental Figure 3. An Additional Biological Replicate Showing phyA Mediating Far-Red Light Promotion of the Phosphorylation of BES1 Protein.**


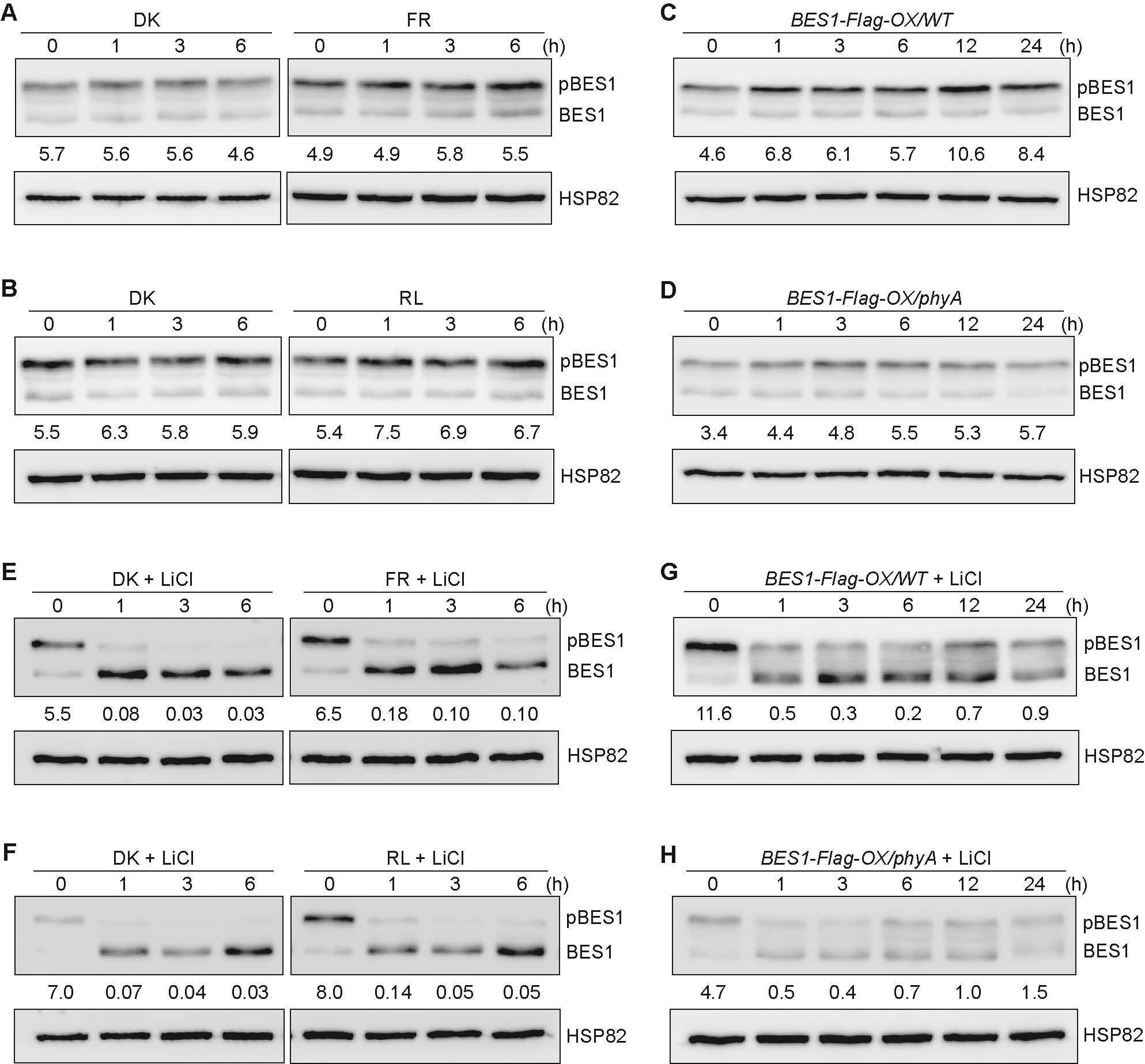


**(A,B)** Western blot assays showing far-red and red lights promotion of phosphorylation of BES1-Flag protein. *BES1-Flag-OX*/WT seedlings were grown in darkness for 5 d, and then exposed to far-red­ light (FR, 10 μmol/m^2^/s) **(A)** or red­ light (R, 50 μmol/m^2^/s) **(B)** or adapted in the dark (DK) for the indicated lengths of time. In the Figure 3, pBES1 and BES1 denote the phosphorylated and dephosphorylated BES1, respectively. The ratio of phosphorylated to dephosphorylated was quantified using Image J and shown below each lane.

**(C,D)** Western blot assays showing that phyA mediates far-red light promotion of phosphorylation of BES1-Flag protein. *BES1-Flag-OX*/WT **(C)** and *BES1-Flag-OX/phyA* **(D)** seedlings were grown in darkness for 5 d, and then exposed to the far-red­ light (10 μmol/m^2^/s) for the indicated lengths of time.

**(E,F)** Western blot assays showing the effects of BIN2 on far-red and red lights promotion of the phosphorylation of BES1-Flag protein. *BES1-Flag-OX*/WT seedlings were grown on MS plates in darkness for 5 d, and then treated with 10 mM LiCl and exposed to far-red­ light (10 μmol/m^2^/s) **(E)** or red­ light (50 μmol/m^2^/s) **(F)** or adapted in the dark for the indicated lengths of time.

**(G,H)** Western blot assays showing the effects of BIN2 on phyA-mediated far-red light promotion of the phosphorylation of BES1-Flag protein. *BES1-Flag-OX*/WT **(G)** and *BES1-Flag-OX/phyA* **(H)** seedlings were grown in darkness for 5 d, and then treated with 10 mM LiCl and exposed to far-red­ light (10 μmol/m^2^/s) for the indicated lengths of time.

**Supplementary Figure 4. The Original Images of Immunoblots Used in This Paper to Prepare Figures.**

**
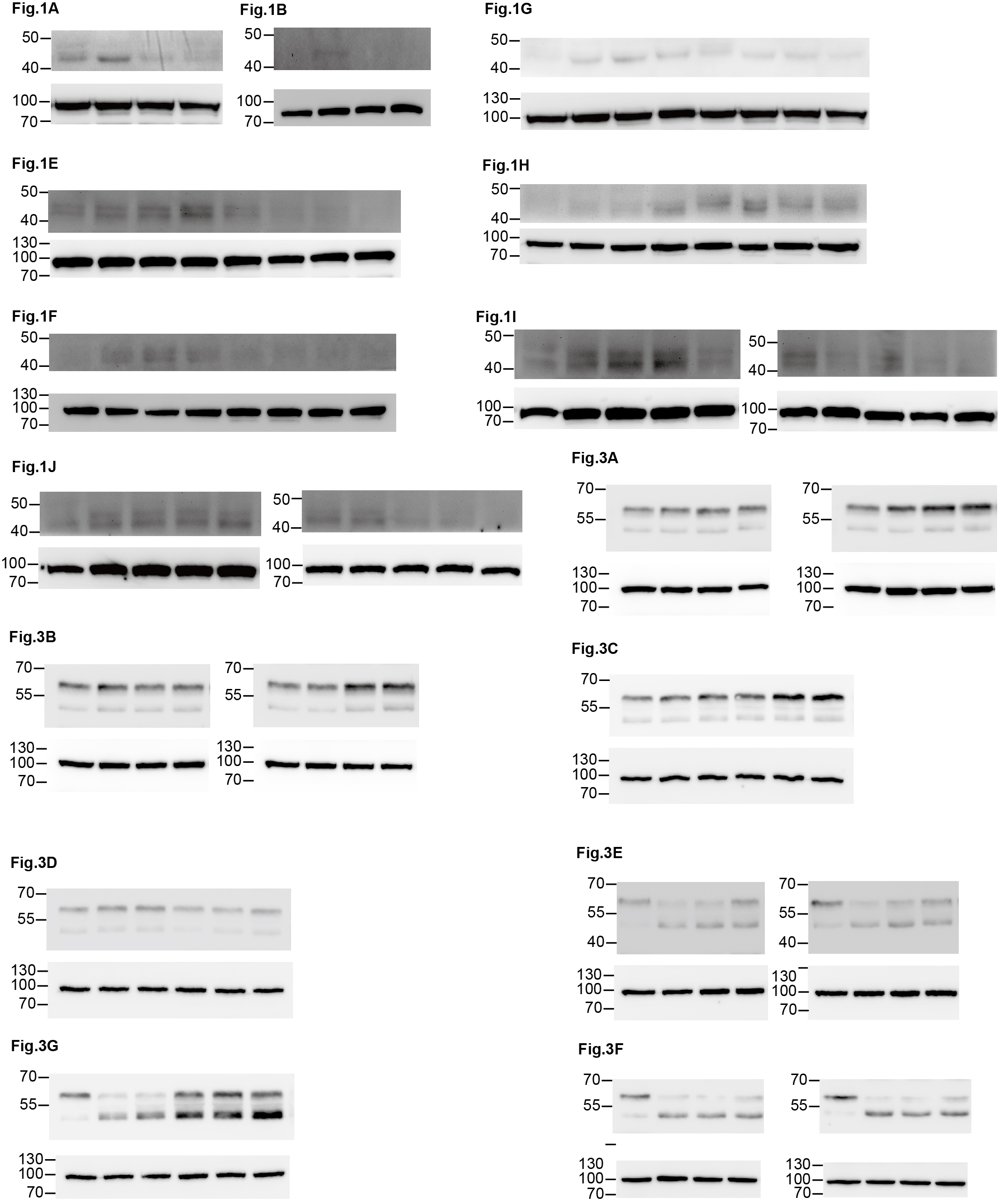
**

**Supplementary Figure 5. The Original Images of Immunoblots Used in This Paper to Prepare Figures.**

**
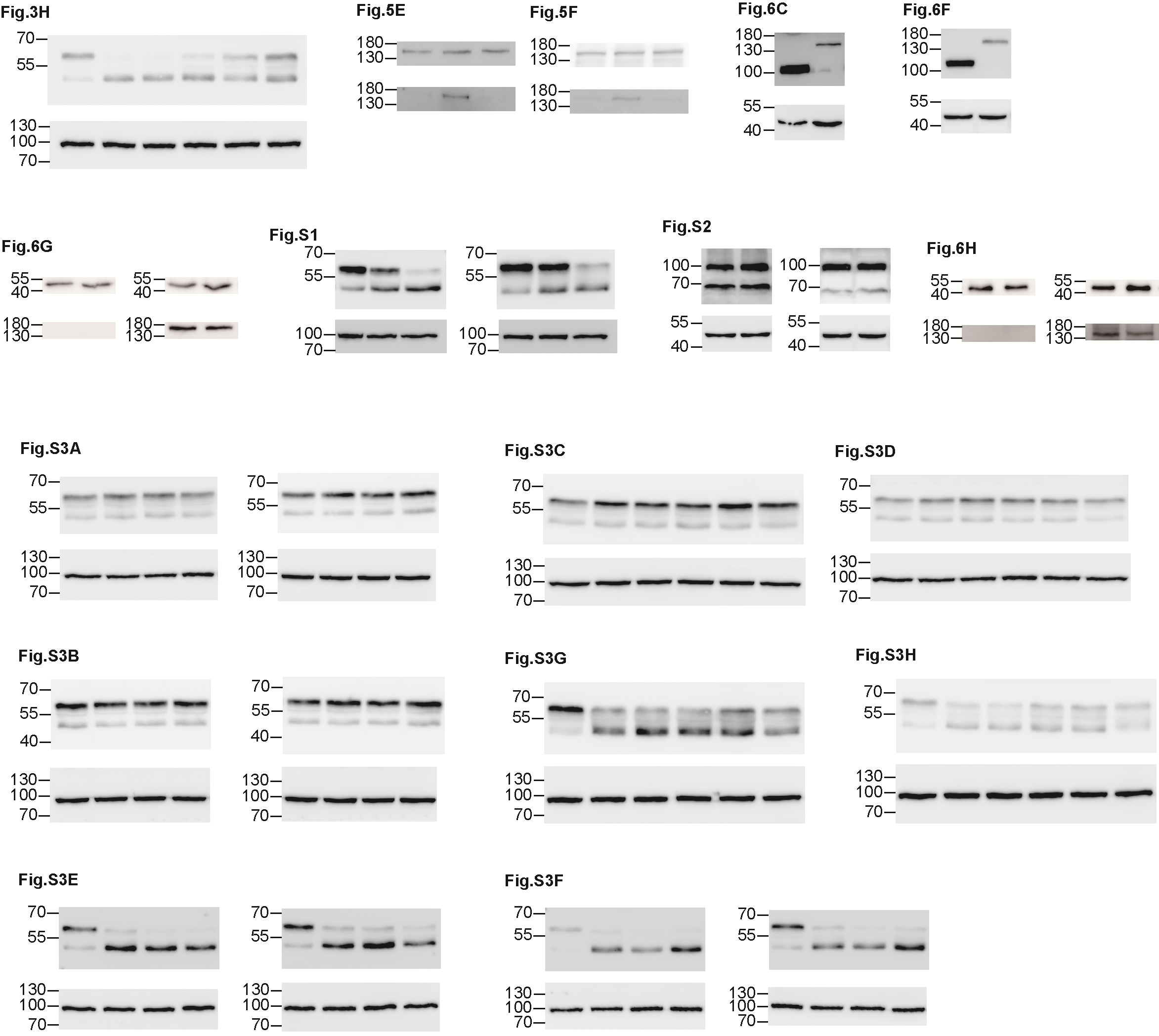
**
