# Supplementary material for: Phytochromes A and B Mediate Light Stabilization of BIN2 to Regulate Brassinosteroid Signaling and Photomorphogenesis in Arabidopsis
Source: Front Plant Sci. 2022 Mar 30;13:865019. doi: 10.3389/fpls.2022.865019 (PMC9005995; doi:10.3389/fpls.2022.865019)
Supplement: Supplementary Table 1 — The primers used in this study. [file Table_1.docx]

**Supplemental Table 1. The Primers Used in This Study**

| **Primer Name** | **Primer Sequence (5'→3')** |
| --- | --- |
| **Primers for Genotyping analysis** | |
| bin2-1-LP | GGCTTTGTCTGAACGAGTTTG |
| bin2-1-RP | AGCTCATATTTGGTGCCACTG |
| phyB-LP | ACGATAGGCTAACTCAGTCTATG |
| phyB-RP | ACTTCCCGTGGTAAAGAAATG |
| phyA-211-F | TTATCCACAGGGTTACAGGG |
| phyA-211-R | GCATTCTCCT TGCATCATCC |
| **Primers for qPCR** | |
| BIN2-qRT-F | TAAGATGCACAGCGCTCGAA |
| BIN2-qRT-R | TGAAGTTGAAGAGAGGCGGG |
| PP2A-qRT-F | TATCGGATGACGATTCTTCGTGCAG |
| PP2A-qRT-R | GCTTGGTCGACTATCGGAATGAGAG |
| **Primers for split-luc assays** | |
| myc-SalⅠ-F | GCGTCGACATGGTGCGACGGTATCGATT |
| myc- BamHⅠ-ns-R | GGACTAGTGGTGAGGTCGCCCAAG |
| phyA- BamHⅠ-F | GGACTAGTATGTCAGGCTCTAGGCCG |
| phyA-SmaⅠ-R | CCCCGGGCTACTTGTTTGCTGCAGCGAGT |
